# Global monitoring of volcanic SO_2_ degassing with unprecedented resolution from TROPOMI onboard Sentinel-5 Precursor

**DOI:** 10.1038/s41598-019-39279-y

**Published:** 2019-02-25

**Authors:** N. Theys, P. Hedelt, I. De Smedt, C. Lerot, H. Yu, J. Vlietinck, M. Pedergnana, S. Arellano, B. Galle, D. Fernandez, C. J. M. Carlito, C. Barrington, B. Taisne, H. Delgado-Granados, D. Loyola, M. Van Roozendael

**Affiliations:** 1 Royal Belgian Institute for Space Aeronomy (BIRA-IASB), Brussels, Belgium; 2 Institut für Methodik der Fernerkundung (IMF), Deutsches Zentrum für Luft und Raumfahrt (DLR), Oberpfaffenhofen, Germany; 3 Department of Space, Earth and Environment, Chalmers University of Technology, Gothenburg, Sweden; 4 Philippine Institute of Volcanology and Seismology (PHIVOLCS), Quezon City, Philippines; 5 Asian School of the Environment, Nanyang Technological University, Singapore, Singapore; 6 Earth Observatory of Singapore (EOS), Nanyang Technological University, Singapore, Singapore; 7 Instituto de Geofísica, Universidad Nacional Autónoma de México (UNAM), Mexico City, Mexico

## Abstract

Over the last four decades, space-based nadir observations of sulfur dioxide (SO_2_) proved to be a key data source for assessing the environmental impacts of volcanic emissions, for monitoring volcanic activity and early signs of eruptions, and ultimately mitigating related hazards on local populations and aviation. Despite its importance, a detailed picture of global SO_2_ daily degassing is difficult to produce, notably for lower-tropospheric plumes, due largely to the limited spatial resolution and coverage or lack of sensitivity and selectivity to SO_2_ of current (and previous) nadir sensors. We report here the first volcanic SO_2_ measurements from the hyperspectral TROPOspheric Monitoring Instrument (TROPOMI) launched in October 2017 onboard the ESA’s Sentinel-5 Precursor platform. Using the operational processing algorithm, we explore the benefit of improved spatial resolution to the monitoring of global volcanic degassing. We find that TROPOMI surpasses any space nadir sensor in its ability to detect weak degassing signals and captures day-to-day changes in SO_2_ emissions. The detection limit of TROPOMI to SO_2_ emissions is a factor of 4 better than the heritage Aura/Ozone Monitoring Instrument (OMI). Here we show that TROPOMI SO_2_ daily observations carry a wealth of information on volcanic activity. Provided with adequate wind speed data, temporally resolved SO_2_ fluxes can be obtained at hourly time steps or shorter. We anticipate that TROPOMI SO_2_ data will help to monitor global volcanic daily degassing and better understand volcanic processes and impacts.

## Introduction

Volcanic emissions of sulfur dioxide can affect significantly atmospheric chemistry and global climate. SO_2_ is a precursor of sulfate aerosols, important for air quality^1^, and sulfuric acid, a compound known to alter local ecosystems, and which can cause damage to aircraft engines^2,3^. Injection of SO_2_ in the upper-troposphere and lower-stratosphere can lead to significant changes on global climate^4^ although the role of modest eruptions on volcanic forcing is only partly understood and is still an important subject of research^5–7^.

Measurement of volcanic SO_2_ degassing is also vitally important for volcano monitoring and to understand the underlying processes that can ultimately lead to an eruption^8,9^, or for following up an ongoing eruption^10^. In this respect, remote sensing of SO_2_ has been widely carried out from ground^11–13^, owing to the ease of SO_2_ detection, due to its strong absorption in the near UV and its low atmospheric background. Satellite nadir SO_2_ measurements with their global daily coverage provide complementary information for unmonitored volcanoes and explosive eruptions. Since the early measurements of SO_2_ from space in the late seventies^14,15^, advances in instrumentation and retrieval techniques, in both ultraviolet (UV) and thermal infrared (TIR) spectral ranges, have helped better understand volcanic processes, pre-eruptive signs or eruption (together with other geophysical data) and assess the impact of volcanic degassing on the atmosphere in general^16^. Global SO_2_ data has been increasingly recognized as a crucial data source and is now being used e.g. for near real-time monitoring of volcanic plumes^17^.

Satellite observations of volcanic SO_2_ emissions has mostly been used for estimation of total masses of SO_2_ emitted during explosive eruptions or from strongly degassing and/or high elevation volcanoes^16,18–21^, and to a lesser extent for the inversion of SO_2_ fluxes^22–27^. For passive degassing volcanoes, which dominate by large the time-averaged global volcanic emission^28^, space-based constraints on SO_2_ fluxes are more difficult to obtain. Typically, the SO_2_ concentrations are lower, the SO_2_ plume can be sub-pixel sized (especially near the source) and it is generally located in the lower troposphere where the measurement sensitivity is less favourable (and where rapid oxidation or wet deposition can also be a complicating factor). For these reasons, the state-of-the art in global SO_2_ emissions monitoring had been obtained using the Aura/Ozone Monitoring Instrument^29^ (OMI), as it combines the advantages of good sensitivity and selectivity to SO_2_ in the lower troposphere (compared to other sensors), and reasonably high nadir spatial resolution of 13 × 24 km^2^. Recently, improved methodology to estimate mean SO_2_ emissions from space measurements has been developed^30^ and applied to 10 years of OMI SO_2_ data over many degassing volcanoes, resulting in the most complete inventory of global volcanic SO_2_ annual emissions^28^ produced so far.

With a spatial footprint of 7 × 3.5 km² (13 times better than OMI, at least), the Sentinel-5 Precursor (S5P) TROPOMI instrument^31^ opens new possibilities in the surveillance of volcanic SO_2_ from space, and in quantifying robustly SO_2_ emission changes on shorter time intervals. SO_2_ clouds are detected globally and mapped with unprecedented detail, and even the weaker SO_2_ degassing plumes are measured nearly on a daily basis. Here, we present the first TROPOMI SO_2_ results and we examine and demonstrate several advantages of high spatial resolution data for volcano monitoring. We investigate the improvement in detection limit and the implication on the increased frequency of detecting weak emissions. We also investigate the potential of TROPOMI to provide high temporal resolution information on SO_2_ emission and compare the obtained results with operational ground-based SO_2_ flux data.

## Data and Methods

### TROPOMI and OMI SO_2_ vertical column data

SO_2_ data used in this study were retrieved from backscattered radiance measurements of TROPOMI using the S5P operational processing algorithm. The retrieval scheme is completely described elsewhere^32,33^, and is only briefly summarized here. The same algorithm was also applied to measurements obtained by the predecessor OMI sensor to produce a scientific dataset^34^ (different from the operational NASA SO_2_ product) for comparison with the presented TROPOMI data. Details on instruments characteristics and retrieval settings are provided in Tables S1 and S2 (Supplementary Material).

SO_2_ slant column densities, representing the effective optical-path integral of SO_2_ concentration, are retrieved by applying differential optical absorption spectroscopy^35^ (DOAS) to ultraviolet spectra using a combination of three fitting windows (312–326 nm: standard, 325–335 nm or 360–390 nm: alternatives to avoid signal saturation for high SO_2_). To cope with possible biases in the spectral retrieval step, a post-processing correction is then applied to produce so-called background corrected slant column densities (SCDs). The final results of the algorithm are the SO_2_ vertical column densities (VCDs), corresponding to the number of SO_2_ molecules in an atmospheric column per unit area (expressed hereafter in Dobson Units [DU] − 1 DU = 2.69 × 10^16^ molecules/cm²). SO_2_ VCDs are obtained from SCDs using conversion factors (air mass factors) that account for changes in measurement sensitivity due to observation geometry, total ozone absorption, clouds, and surface reflectivity. Since the measurement sensitivity also varies with altitude and the height of the emitted plumes is not known a-priori, the SO_2_ VCDs are calculated for three different hypothetical profiles of this gas, corresponding to 1 km thick boxes, at ground level and centered at 7 km and 15 km a.s.l. For illustration purpose, the SO_2_ VCD maps presented in this study correspond to the 7 km product (unless stated otherwise).

For this work, TROPOMI and OMI SO_2_ column data are used for the period from November 2017 to July 2018, for the days when both sensors were simultaneously measuring. The data over this measurement period were exploited to produce statistics on SO_2_ detection rate (hereafter referred as ‘SO_2_ detection frequency’) over certain volcanoes. For this, we apply a simple scheme independently to each satellite data set. First, a selection is performed on satellite pixels with cloud fraction <0.5, solar zenith angle <70° and central across-track positions (TROPOMI rows 50–400 and OMI rows 5–55). Moreover, we exclude OMI data affected by the row anomaly (http:// projects.knmi.nl/omi/research/product/rowanomaly-background.php). Secondly, all selected pixels for a given day and within a 75 km radius around a given volcano are considered (N in total). The pixels with SO_2_ SCD > 3 × SCDE (where SCDE is the uncertainty on the fitted SO_2_ slant column, typically of 0.3 DU for TROPOMI and 0.25 DU for OMI) are counted (Nc) and a detection is considered as plausible if Nc ≥2 and Nc > 0.04 × N. The latter criteria are necessary to avoid false detections and have been determined from statistics of the data over regions not affected by volcanic emissions. Finally, the SO_2_ detection frequency for a given volcano is simply the daily detection score divided by the total number of usable days for the measurement period.

### TROPOMI-based SO_2_ flux estimation

Volcanic SO_2_ emission rates (expressed in kg/s) are estimated from daily maps of TROPOMI SO_2_ VCD applying the well-established traverse technique^22,23,36,37^. For this, a 3° × 3° square centered at a given volcano is used and the pixels with SO_2_ SCD >1 DU (and with at least one neighboring pixel satisfying the same criterion) are selected to delineate the plume for the analysis. After, the coordinates of the pixels considered are used to infer a mean plume direction, via a simple line fit in longitude-latitude axis. At this point, the method needs ancillary information of the SO_2_ plume height and wind speed (representative of the plume altitude), at the location of the volcano and at satellite overpass time. The plume height and wind data are described in the next section. The plume height is used to recalculate SO_2_ VCDs by linear interpolation of the three SO_2_ VCD products (0–1 km, 7 km and 15 km). The wind speed v is assumed to be constant over the length scale of the plume and does not account for local variations of the wind field. It is used to estimate, for each pixel, the time t elapsed since emission (plume age) which is approximated by t = d/v (where d is the distance in plume direction from the pixel center to the volcano). Then, for 0.5 h (Δt) plume age bins, total SO_2_ masses m are calculated by summing up their corresponding SO_2_ VCDs, and finally values for the SO_2_ flux (F = m/Δt) are derived, for 3 h long plume age intervals (hence six values of F in total, from ‘plume traverses’ at increasing distances from the volcano). This approach follows previous studies^22,23^ that have demonstrated the possibility to reconstruct the emission chronology up to several hours before the overpass time using a single satellite SO_2_ image. We estimate the total relative uncertainty of our SO_2_ flux estimates to be about 50%^23^_,_ with a dominant contribution from the uncertainty on the wind.

### SO_2_ flux from ground-based NOVAC observations

Ground-based data is obtained from scanning-DOAS instruments from the Network for Observation of Volcanic and Atmospheric Change (NOVAC) operated by PHIVOLCS/EOS (Mayon) and UNAM (Popocatépetl). NOVAC is a global network of ground-based remote sensors, providing routinely SO_2_ emissions from several degassing volcanoes. The detailed description of the instruments and analysis is given elsewhere^12^, and summarized in Tables S1 and S2. In essence, the method to determine SO_2_ flux is equivalent to the one described for TROPOMI.

Usually 2–3 scanners are installed around a given volcano (3–10 km distance) to cover most directions of plume transport. A full scan takes about 5–10 min to complete, comprising about 50 angular positions. SCDs of SO_2_ are derived from the measured UV spectra using DOAS (Table S2) and VCDs in the plume (relative to atmospheric background) are obtained assuming a straight-optical path through the plume. From the spatial distribution of VCDs, the total amount of molecules of SO_2_ in a cross section of the plume is obtained. This quantity can then be converted into fluxes by multiplication with the plume velocity. The standard relative uncertainty of a single SO_2_ flux measurement from NOVAC depends strongly on measurements conditions^12^, and is estimated to be within 30%.

The NOVAC data set provides high temporal resolution SO_2_ flux measurements and includes also ancillary data such as plume height and wind-speed. The latter data are used as input of the TROPOMI SO_2_ flux retrievals (as described above), to ensure consistency of TROPOMI and NOVAC comparisons. The plume direction and height can be estimated from NOVAC measurements by triangulation, using the angular distributions of SCDs obtained by simultaneous measurements of the different scanners. When only one instrument produces valid measurements at a given time, plume height is assumed to be equal to the difference between the station and volcano summit altitudes and plume direction is derived geometrically. Plume speed for this study is obtained from wind speed data of the NOAA Global Data Assimilation System (GDAS, 1 deg/3 h, accessed through https://www.ready.noaa.gov/) sampled at the coordinates of the volcano summit and interpolated at the measurement time.

## Results and Discussion

### Monitoring of large SO_2_ emissions

**Figure 1:**
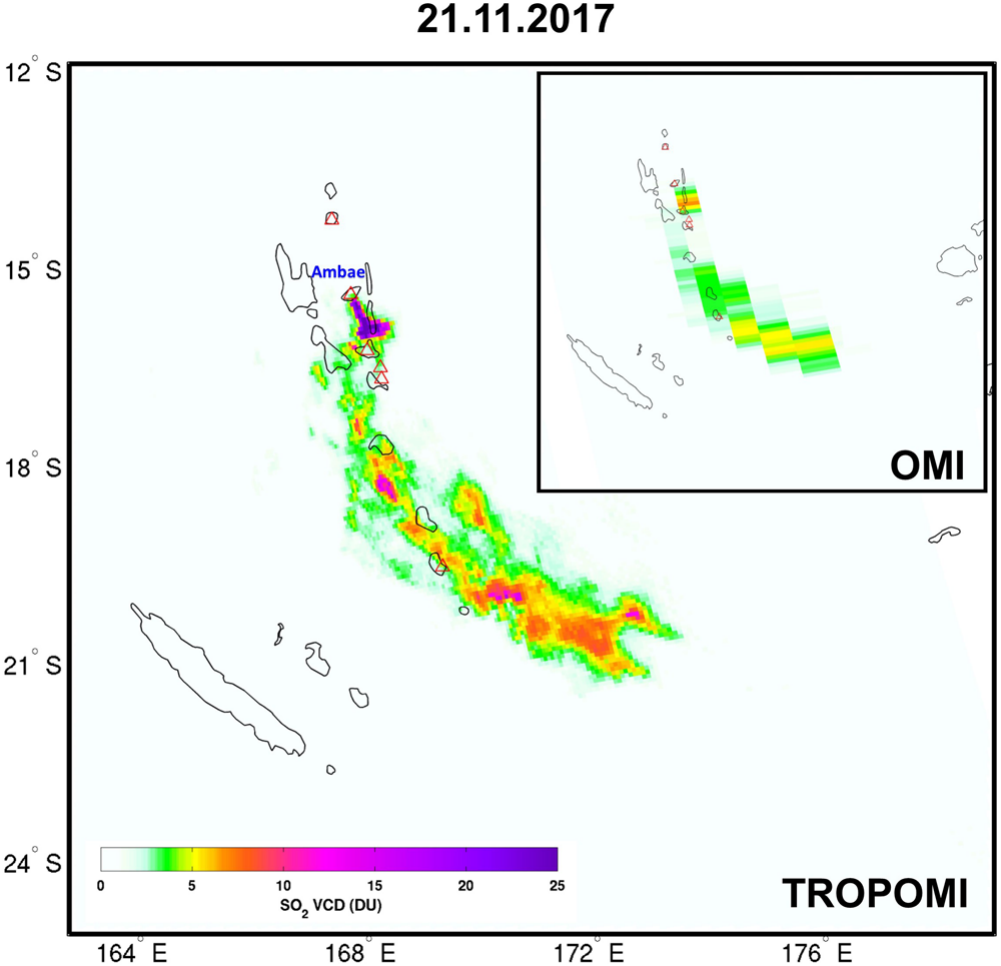
TROPOMI and OMI SO_2_ vertical columns over Vanuatu on November 21, 2017, with SO_2_ emission from Ambae volcano.

During the studied period, several eruptions produced spatially extended SO_2_ plumes that have been successfully detected by TROPOMI. In particular, the Ambae volcano (also known as Aoba) in the archipelago of Vanuatu, continuously emitted copious amounts of SO_2_. Figure 1 shows TROPOMI retrieved SO_2_ vertical columns above Vanuatu on 21 November 2017; the corresponding OMI SO_2_ VCD is shown in inset in Fig. 1 for comparison. The TROPOMI results reveal a stretched SO_2_ plume of ~1000 km length with remarkably more details than OMI SO_2_ columns. Owing to its improved spatial resolution, the TROPOMI data resolves small SO_2_ puffs and local maxima that are likely attributable to short-term variations in volcanic activity. The typical situation with OMI coarse spatial resolution is that the SO_2_ plume covers only a fraction of the pixel, resulting in reduced measured VCD. This is especially important close to the source where the plume horizontal size can be small. This dilution effect is much less pronounced with TROPOMI; the maximum VCD is 34.7 DU for TROPOMI, while it is only of 6.7 DU for OMI. Also note that signal saturation is probably responsible for some differences in the SO_2_ VCDs because the alternative fitting window 325–335 nm is more frequently used for TROPOMI than OMI, notably for the pixels close to the source with elevated SO_2_ columns. As a consequence, TROPOMI measurements are more representative of the volcanic source than OMI data.

Figure 1 demonstrates the spatial resolution at which SO_2_ can be measured from TROPOMI. Considerable information on emission chronology and volcanic processes (highly relevant for volcanologists) can be extracted from measurements downwind. Ongoing efforts to reconstruct SO_2_ emission altitude and flux time-series with up to hourly resolution using inverse modeling approaches^23,24,27,38–40^ will likely benefit from TROPOMI SO_2_ measurements. Enhanced information on short-lived volcanic processes is expected from such analysis, as well as more robust forecasting of dispersion of volcanic clouds (of importance for aviation safety).

### Detection of weak SO_2_ emissions

Whereas TROPOMI has drastically improved spatial resolution compared to OMI (by a factor of 13 better), we find that both sensors have similar spectral quality, e.g. in terms of spectral resolution and radiometric noise. This suggests that TROPOMI also outperforms OMI in detecting weak SO_2_ plumes typically residing in the lower troposphere.

**Figure 2:**
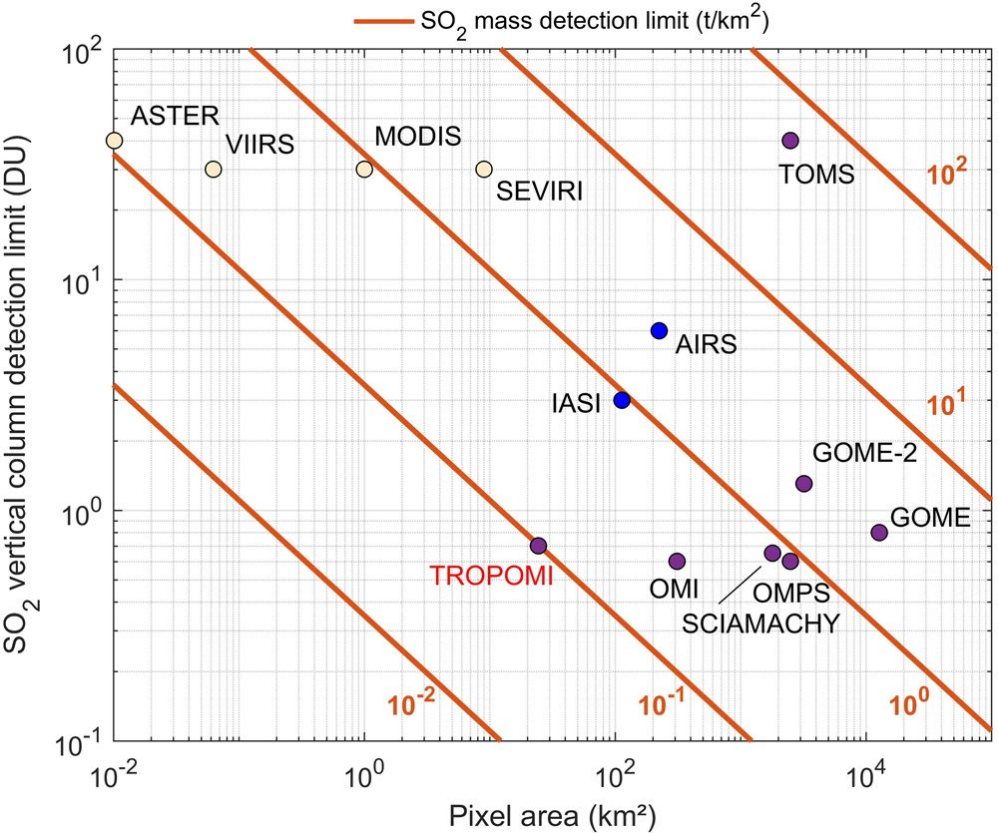
SO_2_ VCD detection limit (at 3-σ level) for a tropospheric plume at 3 km height, as a function of pixel size (in km²) for space nadir sensors with proven capability to detected SO_2_ (values are adapted from the literature and personal communications). Pale orange points are used for thermal infrared imagers: ASTER^44^, VIIRS (detection limit presumably similar as MODIS), MODIS^45,46^, SEVIRI^47^. In blue, the IASI^21,48^ and AIRS^18^ thermal infrared hyperspectral sounders. Purple points are used for UV-VIS instruments: TOMS^49^, GOME^50^, SCIAMACHY^51^, GOME-2^52^, OMPS^53^, OMI^34,53^, and TROPOMI^32,33^ (this study). The orange curves are the corresponding iso-lines of SO_2_ mass detection limit (t/km²).

It is enlightening to compare TROPOMI’s ability of detecting passive degassing plumes not only to OMI, but also to other existing (and past) sensors. Figure 2 compiles typical values of SO_2_ vertical column detection limit (at 3-σ level) for a tropospheric plume at 3 km height, as a function of pixel size (in km²) of a number of established nadir sensors (references are in caption of Fig. 2). UV measurements utilize SO_2_ absorption bands in the wavelength range around 310–340 nm and have good sensitivity to the lower troposphere. Figure 2 illustrates the progress of UV remote sensing of tropospheric SO_2_ since the first measurements performed by TOMS. Although SO_2_ retrieval algorithms have significantly evolved over the last years^34,41^, the increase in information on tropospheric SO_2_ is mainly due to improvement in spatial resolution of operational UV sensors (Fig. 2). TIR measurements of SO_2_ usually exploit absorption bands at 7.3 µm or 8.6 µm. High spatial resolution SO_2_ measurements are being carried out using thermal infrared imagers (such as ASTER and MODIS) but with limited sensitivity and accuracy to lower tropospheric SO_2_, as depicted in Fig. 2. The main reasons are interference by water vapor or volcanic ash (or other types of aerosols), lack of thermal contrast, dependence on variable surface emissivity and low spectral sampling. Better detection limit and selectivity to SO_2_ can be obtained from hyperspectral TIR instruments, like IASI and AIRS, but with larger footprint sizes. To better assess the different instruments, Fig. 2 also features iso-curves of SO_2_ mass detection limit (this quantity scales the SO_2_ VCD detection limit to a unit area of 1 km² and is independent of satellite footprint size). One can see that the ability of TROPOMI to detect volcanic degassing emissions is better than any other space sensor. The only exception is perhaps ASTER, but it is mostly because of its higher spatial resolution of 90 × 90 m². Moreover, ASTER does not allow for global daily coverage (as TROPOMI), and retrievals generally suffer at discriminating SO_2_ from other spectral features.

**Figure 3:**
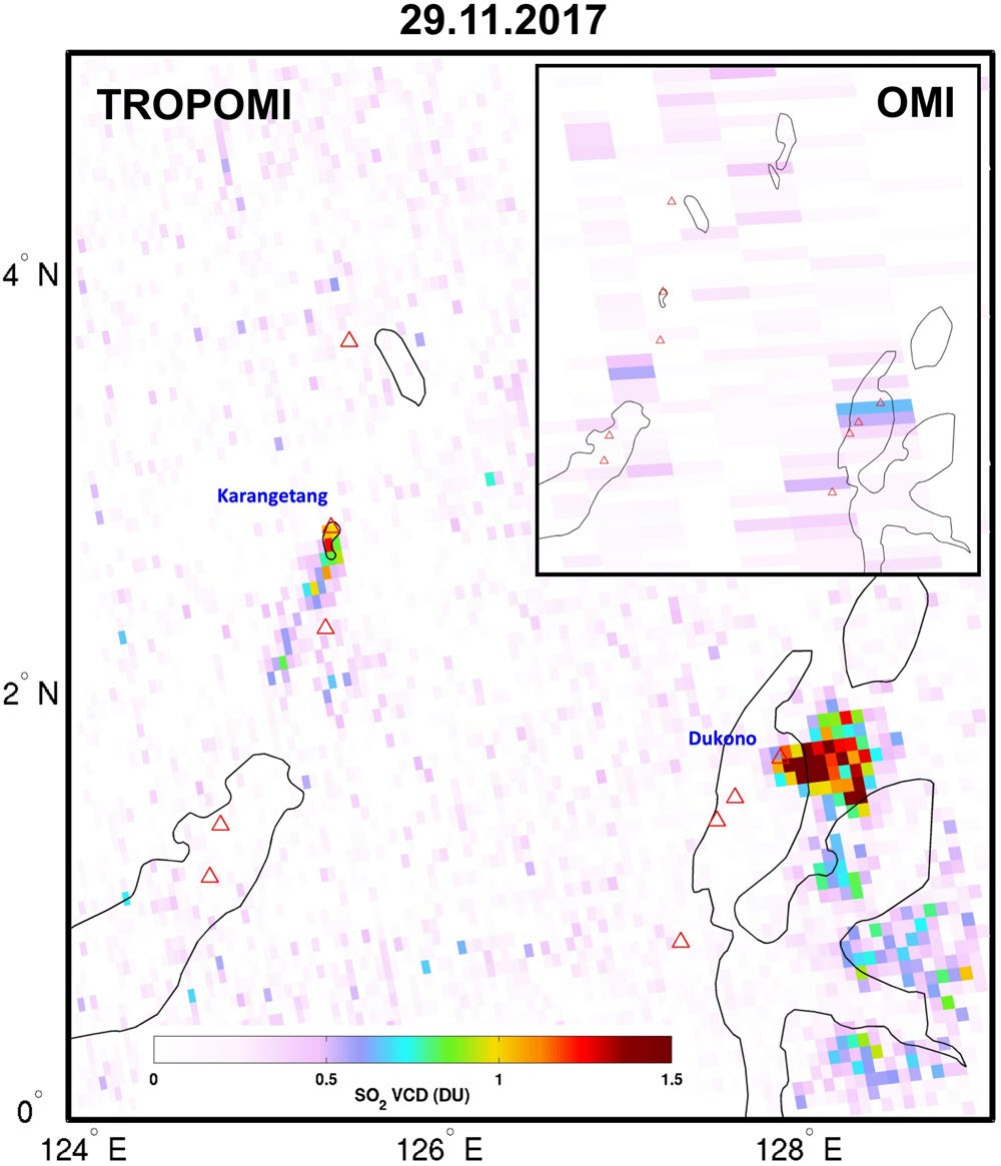
TROPOMI and OMI SO_2_ vertical columns over Indonesia on November 29, 2017, with SO_2_ emission from Karengetang and Dukono volcanoes.

Moreover, we find that TROPOMI has, due to its improved spatial resolution, about 4 times better SO_2_ mass detection limit relative to OMI. This is corroborated by Fig. 3, which shows TROPOMI retrieved SO_2_ vertical columns and corresponding OMI data over Indonesia, on November 29, 2017. On that day, two degassing volcanoes were particularly active in this region, Dukono and Karangetang. While TROPOMI unambiguously measures SO_2_ emissions from both volcanoes, OMI only detects emissions from Dukono (and with much less detail than TROPOMI). The small SO_2_ plume from Karangetang is undetected by OMI because of the coarse spatial resolution of the instrument. The example of Fig. 3 is often observed at other weakly emitting volcanoes worldwide (see Supplementary Material, Figs S1–3).

**Figure 4:**
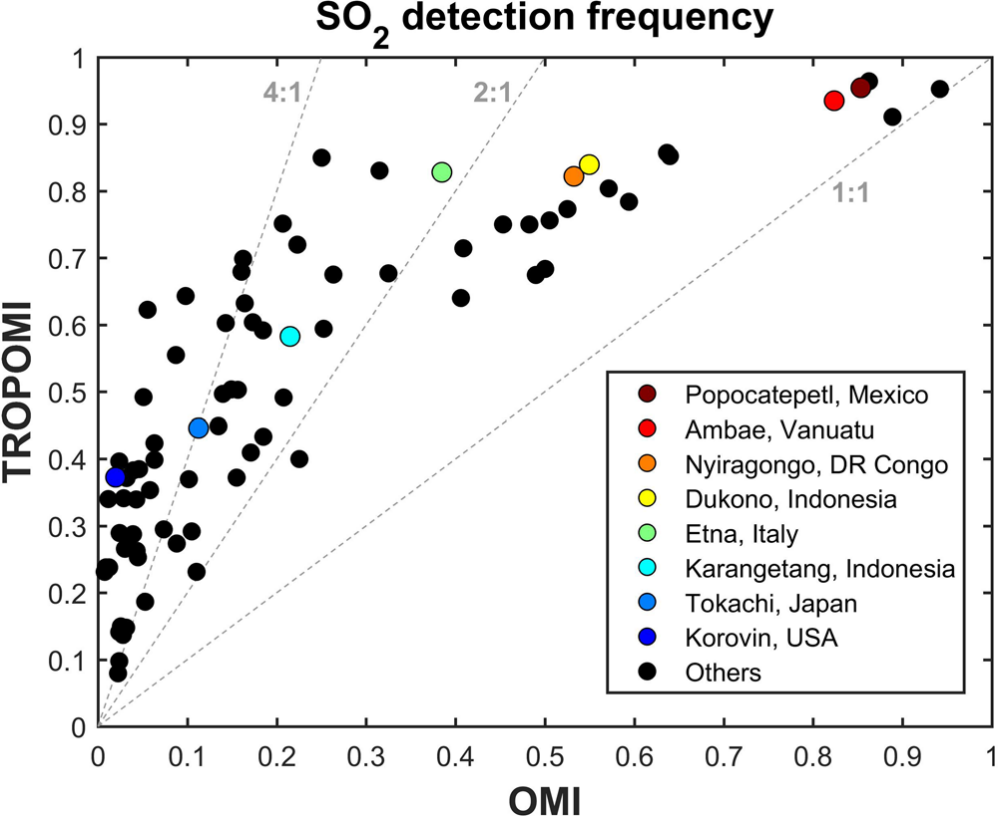
SO_2_ daily detection frequency derived from TROPOMI and OMI data from November 2017 to July 2018, following the method described in section 2. The points correspond to the degassing volcanoes (based on the global inventory)^28^ active during this measurement period. Several volcanoes are identified by a color code. The dashed lines correspond to 1:1, 2:1 and 4:1 lines.

Based on global SO_2_ maps of averaged data over the measurement period (not shown), we have investigated all volcanoes reported in the recent volcanic SO_2_ emissions inventory^28^ and found that 58 degassing volcanoes were unambiguously detected in the TROPOMI measurements for the period from November 2017 to July 2018. Following the method described in section 2, we have calculated the frequency of daily SO_2_ detection for all 58 volcanoes. Figure 4 compares the results for OMI and TROPOMI data. While the presence of the OMI row anomaly data gap is likely to play a role in this picture, we find that TROPOMI is always detecting SO_2_ more frequently than OMI, as expected from improved spatial resolution. A striking feature of this analysis is that the SO_2_ detection frequency depends a lot on the volcano considered. For strong SO_2_ emitters (e.g., Popocatépetl, Ambae), SO_2_ columns are largely above the limit of detection, and both sensors have comparable performances in detecting SO_2_, with detection frequency values close to 1. Hence, for these cases, TROPOMI and OMI will detect SO_2_ on a nearly daily basis (and the advantage of TROPOMI resides mainly in providing more detailed data, as illustrated in Fig. 1). Conversely, for volcanoes with lower emission strengths, the situation is different, and we find that TROPOMI confidently detects SO_2_ plumes 2–4 times more frequently than OMI. For example, for the Karategang case of Fig. 3, TROPOMI detects SO_2_ about 3/5 of the time while it is detected rather occasionally by OMI. For volcanoes like Korovin and Tokachi, with emissions of 100–200 t SO_2_/d (according to the inventory)^28^, SO_2_ columns are below or close to the detection limit of TROPOMI and the detection frequency values are about 40% (albeit with large uncertainties). Those plumes are quite rarely detected (or not at all) in daily OMI SO_2_ images, and only appears in the global OMI inventory because of the pixels binning approach used^28^.

More work is needed to better quantify the performance of TROPOMI data as a function of SO_2_ emission flux but the conclusion of the analysis above is that monitoring of volcanic plumes nearly on a (clear-sky) daily basis is possible with TROPOMI, for an increasing number of (weakly emitting) volcanoes. This finding is an important step forward in monitoring volcanic degassing at global scale, and to improve budgets of global volcanic emission which is largely dominated by quiescently degassing activity^28^.

### SO_2_ flux retrievals

As abovementioned, the higher-spatial resolution of TROPOMI combined with its increased sensitivity offer the possibility to study more frequently and robustly short-term changes in degassing patterns (at daily or sub-daily resolution), by extracting information on SO_2_ fluxes from the satellite SO_2_ imagery itself. Here we demonstrate the potential of TROPOMI to provide hourly to sub-hourly information on SO_2_ emission rates, hence at a frequency beyond the revisiting time of one day of the satellite. Let us consider a volcanic SO_2_ plume at a constant height being advected by a steady horizontal wind of 5 m/s oriented in the TROPOMI across-track direction i.e. a nearly zonal wind, which is a common situation for many degassing volcanoes. The across-track dimension of TROPOMI being 3.5 km, each satellite pixel is in principle representative for roughly 12 minutes of emission. Comparatively, OMI with an across-track dimension of at best 24 km has a time resolution limit of about 80 minutes. Noteworthy and regardless of time resolution considerations, another decisive advantage of TROPOMI for inferring SO_2_ fluxes compared to OMI, is that it resolves and represents much better the SO_2_ plume near the source, which is typically constrained to a narrow horizontal extent for passive degassing volcanoes.

**Figure 5:**
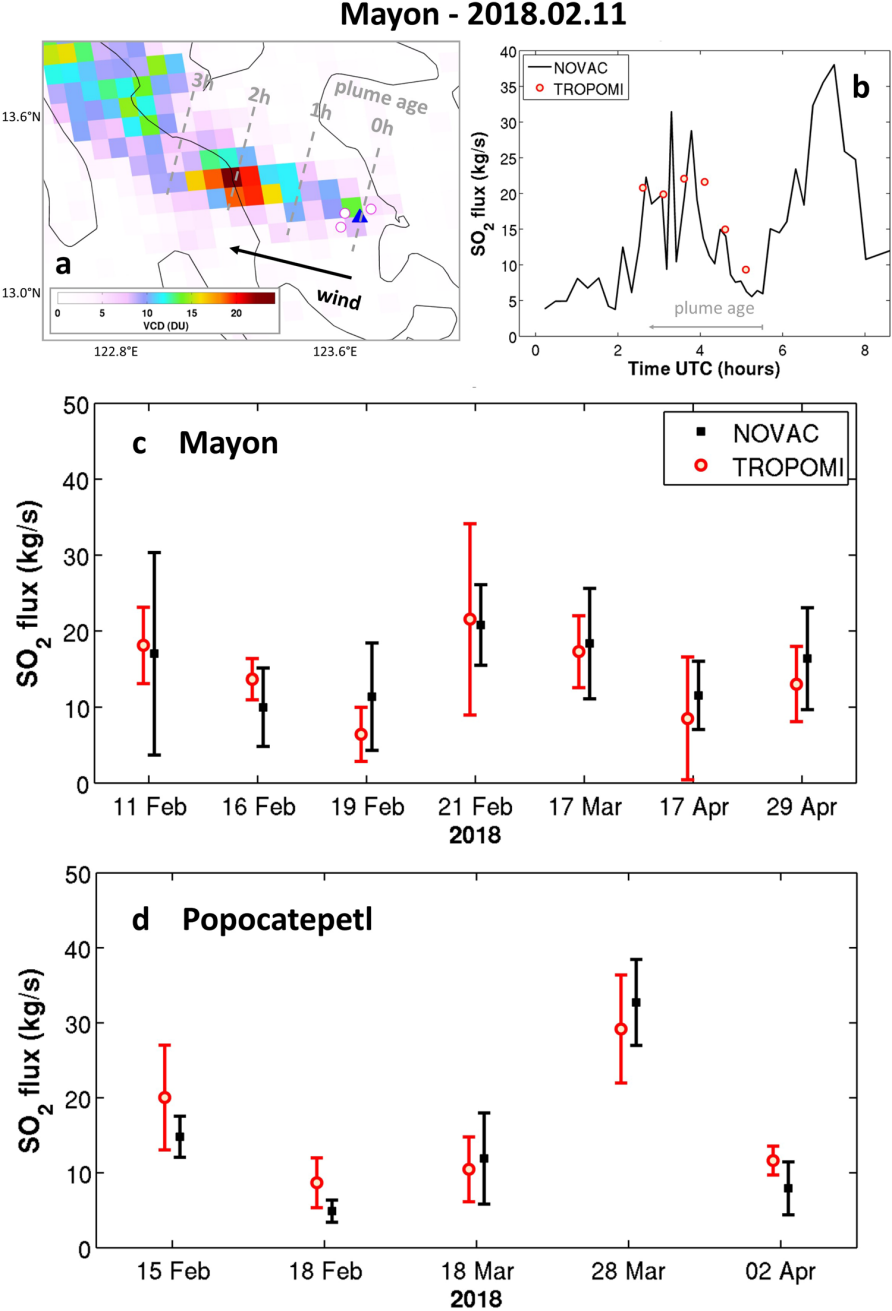
Panels a and b illustrate the TROPOMI SO_2_ flux retrieval technique for the case of Mayon (13.26°N, 123.68°E) on February 11, 2018. (Panel a) TROPOMI SO_2_ VCDs (DU) over the island of Luzon (Philippines), the Mayon volcano is symbolized by a blue triangle, the locations of the NOVAC instruments are marked by magenta circles. From the plume direction and wind speed at plume altitude, a plume age (expressed in hour) is assigned to each TROPOMI pixel. (Panel b) TROPOMI SO_2_ flux (kg/s) obtained at half-hourly sampling from the traverses downwind (hence back in time as illustrated by the gray arrow representing the plume age) and comparison with NOVAC SO_2_ time-series. (Panels c,d) SO_2_ fluxes retrieved from selected days of TROPOMI data for February-April 2018 period, for Mayon and Popocatépetl (19.02°N, 98.63°W) respectively, and estimations from NOVAC measurements. Mean SO_2_ fluxes and standard deviation are depicted for each day.

To validate our approach, we have applied the technique outlined in section 2 to two volcanoes well monitored from the ground, Mayon (Philippines) and Popocatépetl (Mexico), and part of NOVAC. For both volcanoes, we selected the best scenes, i.e. clear-sky satellite overpasses for which TROPOMI detects well-shaped plumes originating from the volcanoes, meaning plumes that are narrow, non-bifurcated and following a main direction, due to stable winds. These conditions are required for validity of the SO_2_ flux retrieval method^23^. An example for Mayon on February 11, 2018 is given in Fig. 5a,b. One can see that the TROPOMI SO_2_ data downwind of the volcano allows the reconstruction of the SO_2_ emission rate (here at half-hourly sampling) up to three hours before the satellite time of observation (~5h30 UTC). This enables identifying short-term changes in SO_2_ degassing, as obvious from the excellent agreement between NOVAC and TROPOMI SO_2_ flux time-series (Fig. 5b). Such results can only be obtained from space using TROPOMI (owing to the combination of high-spatial resolution and high detection limit; Fig. 2).

Note that further application of the SO_2_ flux retrieval technique to other cases is in general not as good as in Fig. 5b for the SO_2_ flux reconstruction. This reflects the limitations in the inversion techniques (both from space and ground) and probably also differences in air mass sampling. Nevertheless, for daily averaged values, the TROPOMI-derived SO_2_ emissions agree very well with the NOVAC estimates, as shown in Fig. 5c,d for a limited number of days (for the February-April 2018 period), for Mayon and Popocatépetl volcanoes, respectively. This corroborates and validates the TROPOMI SO_2_ results and the ability of the instrument to track changes in SO_2_ flux at (sub-)daily time scale^42^.

## Conclusions

We have presented the first SO_2_ vertical column results retrieved from TROPOMI spectral measurements using the S5P operational processing algorithm. With a spatial footprint of 7 × 3.5 km² combined with excellent radiometric characteristics, TROPOMI profiles itself as the best hyperspectral UV imager in space, and we demonstrate in this paper the exceptional level of detail and sensitivity at which SO_2_ emitted by volcanoes can be measured. Compared to other satellite-based nadir sensors, TROPOMI is arguably the best satellite instrument for daily tracking of small emission plumes at a global scale. The limit of detection for SO_2_ emissions is a factor of 4 better with TROPOMI than with its predecessor OMI, and we showed that several volcanic SO_2_ sources that were detectable rather irregularly (and to some extent because of the OMI row anomaly) can now be monitored with TROPOMI on a near-daily basis. We have also investigated the potential of TROPOMI to provide information on volcanic SO_2_ flux by comparing the results to data from the NOVAC network, and found that day-to-day changes in SO_2_ emission are well reproduced and that –for the most favorable conditions– the inversion of SO_2_ flux is possible with a time-resolution of less than to an hour.

The TROPOMI SO_2_ product introduced here will constitute a key data source for global volcanic surveillance in the next years. In particular, for weakly emitting and poorly or non-monitored volcanoes, TROPOMI will provide a unique source of data for tracking changes in degassing patterns and, in combination with other types of data (e.g., on seismicity or ground deformation), for detecting early signs of eruption. Efforts to further validate and characterize the product quality at well-equipped volcanic sites are therefore critically needed. We anticipate that innovative techniques based on TROPOMI data will be developed in the future to estimate SO_2_ fluxes more systematically and at more volcanoes. This will improve the study of global volcanic processes and the interplay between volcanic emissions and the atmosphere, and open new possibilities for service applications.

## Supplementary information

Supplementary information

## Data Availability

The operational TROPOMI products are generated by DLR on behalf of ESA and EU Copernicus; the near-real-time products are available in less than three hours after sensing. The TROPOMI SO_2_ products will be publicly available in October 2018, and a detailed description of the SO_2_ product format is given elsewhere^43^. The OMI SO_2_ research data set is available upon request from Nicolas Theys (theys@aeronomie.be). Access to the NOVAC data is permitted with consent of the respective volcanological observatories owning the source instruments, according to their internal policies for data administration. Please refer the author list for contact details.

## References

[1] Chin M, Jacob D (1996). Anthropogenic and natural contributions to tropospheric sulfate: A global model analysis. J. Geophys. Res..

[2] Prata AJ (2009). Satellite detection of hazardous volcanic clouds and the risk to global air traffic. Nat. Hazards.

[3] Carn SA, Krueger AJ, Krotkov NA, Yang K, Evans K (2009). Tracking volcanic sulfur dioxide clouds for aviation hazard mitigation. Natural Hazards.

[4] Robock A (2000). Volcanic eruptions and climate. Rev. Geophys..

[5] Solomon S (2011). The persistently Variable “Background” Stratospheric Aerosol Layer and Global Climate Change. Science.

[6] Vernier JP (2011). Major influence of tropical volcanic eruptions on the stratospheric aerosol layer during the last decade. Geophys. Res. Lett..

[7] Santer BD (2014). Volcanic contribution to decadal changes in tropospheric temperature. Nat. Geosci..

[8] Shinohara H (2008). Excess degassing from volcanoes and its role on eruptive and intrusive activity. Rev. Geophys..

[9] Oppenheimer C, Scaillet B, Martin S (2011). Sulfur degassing from volcanoes: source conditions, surveillance, plume chemistry and Earth system impacts. Reviews in Mineralogy and Geochemistry, v..

[10] Delgado Granados H, Piedad-Sánchez N, Cárdenas González L (2001). Sulfur dioxide emissions from Popocatépetl volcano (Mexico): case study of a high-flux passively-degassing erupting volcano. J. Volcanol. Geotherm. Res..

[11] Stix, J., Williams-Jones, G. & Hickson, C. Applying the COSPEC at Active Volcanoes. In: Williams-Jones, G., Stix, J. & Hickson, C. (eds) The COSPEC Cookbook: Making SO_2_ Measurements at Active Volcanoes. *IAVCEI*, *Methods in Volcanology*, 1, 121–167 (2008).

[12] Galle B (2010). Network for Observation of Volcanic and Atmospheric Change (NOVAC)—A global network for volcanic gas monitoring: Network layout and instrument description. J. Geophys. Res..

[13] Fickel M, Delgado-Granados H (2017). On the use of different spectal windows in DOAS evaluations: Effects on the estimation of SO2 emission rate and mixing ratios during strong emission of Popocatépetl volcano. Chemical Geology.

[14] Krueger AJ (1983). Sighting of El Chichon sulfur dioxide clouds with the Nimbus 7 total ozone mapping spectrometer. Science.

[15] Prata, A., Rose, W., Self, S. & O’Brien, D. Global, long-term sulphur dioxide measurements from TOVS data: A new tool for studying explosive volcanism and climate, in: Volcanism and the Earth’s Atmosphere, edited by: Robock, A. & Oppenheimer, C., vol. 139 of *Geophys. Monogr*., 75–92, AGU, Washington, DC (2003).

[16] Carn SA, Clarisse L, Prata AJ (2016). Multi-decadal satellite measurements of global volcanic degassing. J. Volcanol. Geotherm. Res..

[17] Brenot H (2014). Support to Aviation Control Service (SACS): an online service for near real-time satellite monitoring of volcanic plumes. Nat. Hazards Earth Syst. Sci..

[18] Prata A, Bernardo C (2007). Retrieval of volcanic SO2 column abundance from Atmospheric Infrared Sounder data. J. Geophys. Res..

[19] Corradini S, Merucci L, Prata AJ, Piscini A (2010). Volcanic ash and SO_2_ in the 2008 Kasatochi eruption: Retrievals comparison from different IR satellite sensors. J. Geophys. Res..

[20] Krotkov NA, Schoeberl MR, Morris GA, Carn S, Yang K (2010). Dispersion and lifetime of the SO2 cloud from the August 2008 Kasatochi eruption. J. Geophys. Res..

[21] Clarisse L (2012). Retrieval of sulphur dioxide from the infrared atmospheric sounding interferometer (IASI). Atmos. Meas. Tech..

[22] Merucci L, Burton MR, Corradini S, Salerno GG (2011). Reconstruction of SO_2_ flux emission chronology from space-based measurements. J. Volcanol. Geotherm. Res..

[23] Theys N (2013). Volcanic SO_2_ fluxes derived from satellite data: a survey using OMI, GOME-2, IASI and MODIS. Atmos. Chem. Phys..

[24] Moxnes ED (2014). Separation of ash and sulfur dioxide during the 2011 Grímsvötn eruption. J. Geophys. Res. Atmos..

[25] Campion R (2014). New lava lake at Nyamuragira volcano revealed by combined ASTER and OMI SO2 measurements. Geophys. Res. Lett..

[26] McCormick B, Popp C, Andrews B, Cottrell E (2015). Ten years of satellite observations reveal highly variable sulphur dioxide emissions at Anatahan Volcano, Mariana Islands. J. Geophys. Res. Atmos..

[27] Pardini, F., Burton, M., Arzilli, F., La Spina, G. & Polacci, M. SO2 emissions, plume heights and magmatic processes inferred fromsatellite data: The 2015 Calbuco eruptions. *J*. *Volcanol*. *Geotherm*. *Res*., 10.1016/j.jvolgeores.2018.08.001 (2018).

[28] Carn SA, Fioletov VE, McLinden CA, Li C, Krotkov NA (2017). A decade of global volcanic SO_2_ emissions measured from space. Sci. Rep..

[29] Levelt PF (2006). The Ozone Monitoring Instrument. IEEE Trans. Geo. Rem. Sens..

[30] Fioletov V (2016). A global catalogue of large SO2 sources and emissions derived from the Ozone Monitoring Instrument. Atmos. Chem. Phys..

[31] Veefkind, J. P. *et al*. TROPOMI on the ESA Sentinel-5 Precursor: A GMES mission for global observations of the atmospheric composition for climate, air quality and ozone layer applications. *Remote Sensing of Environment*, 10.1016/j.rse.2011.09.02710.1016/j.rse.2011.09.027 (2012).

[32] Theys N (2017). Sulfur dioxide operational retrievals from TROPOMI onboard Sentinel-5 Precursor: Algorithm Theoretical Basis. Atmos. Meas. Tech..

[33] Theys, N. *et al*. S5P ATBD of the Sulfur dioxide product, available at: http://www.tropomi.eu/data-products/level-2-products (2018).

[34] Theys, N. *et al*. Sulfur dioxide vertical column DOAS retrievals from the Ozone Monitoring Instrument: Global observations and comparison to ground‐based and satellite data. *J*. *Geophys*. *Res*. *Atmos*., 120, 10.1002/2014JD022657 (2015**)**.

[35] Platt, U. & Stutz, J. Differential Optical Absorption Spectroscopy (DOAS), Principle and Applications. ISBN 3-340-21193-4, Springer Verlag, Heidelberg (2008).

[36] Urai M (2004). Sulfur dioxide flux estimation from volcanoes using Advanced Spaceborne Thermal Emission and Reflection Radiometer: A case study of Miyakejima volcano, Japan. J. Volcanol. Geotherm. Res..

[37] Pugnaghi S, Gangale G, Corradini S, Buongiorno MF (2006). Mt. Etna sulfur dioxide fluxmonitoring using ASTER-TIR data and atmospheric observations. J. Volcanol. Geotherm. Res..

[38] Eckhardt, S., Prata, A. J., Seibert, P., Stebel, K. & Stohl, A. Estimation of the vertical profile of sulfur dioxide injection into the atmosphere by a volcanic eruption using satellite column measurements and inverse transport modeling. *Atmos*. *Chem*. *Phys*. **8**, 3881–3897, http://www.atmos-chem-phys.net/8/3881/2008/ (2008).

[39] Boichu M (2015). Temporal variations of flux and altitude of sulfur dioxide emissions during volcanic eruptions: implications for long-range dispersal of volcanic clouds. Atmos. Chem. Phys..

[40] Kristiansen, N. *et al*. Improving Model Simulations of Volcanic Emission Clouds and Assessing Model Uncertainties, Natural Hazard Uncertainty Assessment: Modeling and Decision Support. Edited by: Karin Riley, Peter Webley, Matthew Thompson, pp105–124, 10.1002/9781119028116.ch8 (2017).

[41] Li, C., Joiner, J., Krotkov, N. A. & Bhartia, P. K. A fast and sensitive new satellite SO_2_ retrieval algorithm based on principal component analysis: application to the ozone monitoring instrument. *Geophys*. *Res*. *Lett*., 40, 10.1002/2013GL058134 (2013).

[42] Queiβer, M. *et al*. TROPOMI enables high resolution SO_2_ flux observations from Mt. Etna, Italy, and beyond. *Sci*. *Rep*. (accepted) (2019). 10.1038/s41598-018-37807-w PMC6353956 30700778

[43] Pedergnana, M., Loyola, D., Apituley, A., Sneep, M. & Veefkind, P. S5P PUM of Sulfur dioxide, available at: https://sentinel.esa.int/documents/247904/2474726/Sentinel-5P-Level-2-Product-User-Manual-Sulphur-Dioxide (2018).

[44] Campion R (2010). Measuring volcanic degassing of SO_2_in the lower troposphere with ASTER band ratios. J. Volcanol. Geotherm. Res..

[45] Watson I (2004). Thermal infrared remote sensing of volcanic emissions using the moderate resolution imaging spectroradiometer. J. Volcanol. Geoth. Res..

[46] Corradini S, Merucci L, Prata AJ (2009). Retrieval of SO_2_ from thermal infrared satellite measurements: correction procedures for the effects of volcanic ash. Atmos. Meas. Tech..

[47] Prata A, Kerkmann J (2007). Simultaneous retrieval of volcanic ash and SO_2_ using MSG-SEVIRI measurements. Geophys. Res. Lett..

[48] Bauduin S (2016). Retrieval of near-surface sulfur dioxide (SO_2_) concentrations at a global scale using IASI satellite observations. Atmos. Meas. Tech..

[49] Krueger AJ (1995). Volcanic sulfur dioxide measurements from the Total Ozone Mapping Spectrometer (TOMS) Instruments. J. Geophys. Res..

[50] Eisinger M, Burrows JP (1998). Tropospheric sulfur dioxide observed by the ERS-2 GOME instrument. Geophys. Res. Lett..

[51] Afe OT, Richter A, Sierk B, Wittrock F, Burrows JP (2004). BrO emissions from volcanoes: a survey using GOME and SCIAMACHY measurements. Geophys. Res. Lett..

[52] Rix M (2012). Volcanic SO_2_, BrO and plume height estimations using GOME-2 satellite measurements during the eruption of Eyjafjallajökull in May 2010. J. Geophys. Res..

[53] Li C (2017). New-generation NASA Aura Ozone Monitoring Instrument (OMI) volcanic SO_2_ dataset: Algorithm description, initial results, and continuation with the Suomi-NPP Ozone Mapping and Profiler Suite (OMPS). Atmos. Meas. Tech..

