# Supplementary material for: Global monitoring of volcanic SO2 degassing with unprecedented resolution from TROPOMI onboard Sentinel-5 Precursor
Source: Sci Rep. 2019 Feb 25;9:2643. doi: 10.1038/s41598-019-39279-y (PMC6390096; doi:10.1038/s41598-019-39279-y)
Supplement: Supplementary file 1 — Supplementary information [file 41598_2019_39279_MOESM1_ESM.pdf]

Supplementary information

N. Theys, P. Hedelt, I. De Smedt, C. Lerot, H. Yu, J. Vlietinck, M. Pedergnana, S. Arellano, B. Galle, D. Fernandez, C.J.M. Carlito, C. Barrington, B. Taisne, H. Delgado-Granados, D. Loyola, M. Van Roozendael, Global monitoring of volcanic SO_2 degassing with unprecedented resolution from TROPOMI onboard Sentinel-5 Precursor.

Supplementary Tables

The table S1 summarizes the technical characteristics of the TROPOMI, OMI and NOVAC instruments. The table S2 details the settings used for the retrieval of SO_2 slant column densities and SO_2 fluxes from TROPOMI and NOVAC data.

Supplementary Figures

The following figures (Figs S1-3) show daily regional maps of SO_2 vertical columns from TROPOMI and OMI for selected volcanic sources.

Characteristic	TROPOMI	OMI	NOVAC
Method	Push-broom nadir imaging spectrometer	Push-broom nadir imaging spectrometer	Dual-beam scanning grating spectrometer
Orbit or location	Polar, sun-synchronous (orbit 824 km)	Polar, sun-synchronous (orbit 705 km)	Stationary (3–10 km from volcano)
Sampling interval	Daily (asc. node 13:30 LT)	Daily (asc. node 13:45 LT)	2–10 min (during daylight)
Spectral range / nm	270–495 (UV-UVIS bands)	270–504 (UV1/2-VIS bands)	280–420 (filter cutoff at 360)
Average spectral resolution / nm (FWHM)	0.44–0.6	0.42–0.63	0.6
Average spectral sampling / nm $pixel^{-1}$	0.07–0.22	0.14–0.33	0.07
Swath IFOV (scan range) / deg	108 (2600 km on ground)	115 (2600 km on ground)	>180 (scan range, 1.8 step)
Flight IFOV / deg	0.3 (3.5 km on ground)	2 (12 km on ground)	0.46 (circular FOV)
Spatial sampling / km^2	7×3.5	13×24	NA
Detector	2D (1024×1024 pixels) back-illuminated CCD	2D (576×780 pixels) back-illuminated CCD	1D (2048 pixels) coated CCD
Period of operation (start)	From November 2017	From July 2004	From Mar 2004
Spatial coverage	Global, daily	Global, daily (with gaps since 2008, due to row anomaly)	41 volcanoes
Calibration	On-flight and on-ground	On-flight and on-ground	Periodic and modelled
Reference	[1]	[2]	[3]

Table S1. Technical characteristics of the TROPOMI, OMI and NOVAC instruments.

Evaluation setting	TROPOMI	NOVAC
Spectral range / nm	312-326 (w1), 325-335 (w2);<br>360-390 (w3)	310-325
Absorption cross section	SO_2 (203 K), O_3 (228 K, 243 K), pseudo O_3 (lambda and square), Ring (modelled at 20° & 87° SZA);<br>SO_2 (203 K), NO_2 (220 K), O_2-O_2; Ring effect (modeled)	SO_2 (293 K), O_3 (228 K), Ring effect (modelled)
Broad-band extinction	5th order polynomial;<br>4th order polynomial	5th order polynomial
Intensity offset	Linear offset	Constant offset,<br>dark spectrum measured
Spectral shift and stretch	Fitted	Fitted or from Fraunhofer spectrum
Reference spectrum	Measured for each detector row over a clean region in the equatorial Pacific Ocean, averaged for 4 days moving window.	Measured for each scan at zenith, then scan baseline is compensated
Plume integration	Sum over scene, based on assumed weighting function and concentration profile	Sum over scans, based on measured plume direction and height
Flux calculation	Based on traverse method	Based on scanning/traverse method

Table S2. Standard evaluation settings for SO_2 flux retrieval from TROPOMI and NOVAC data.

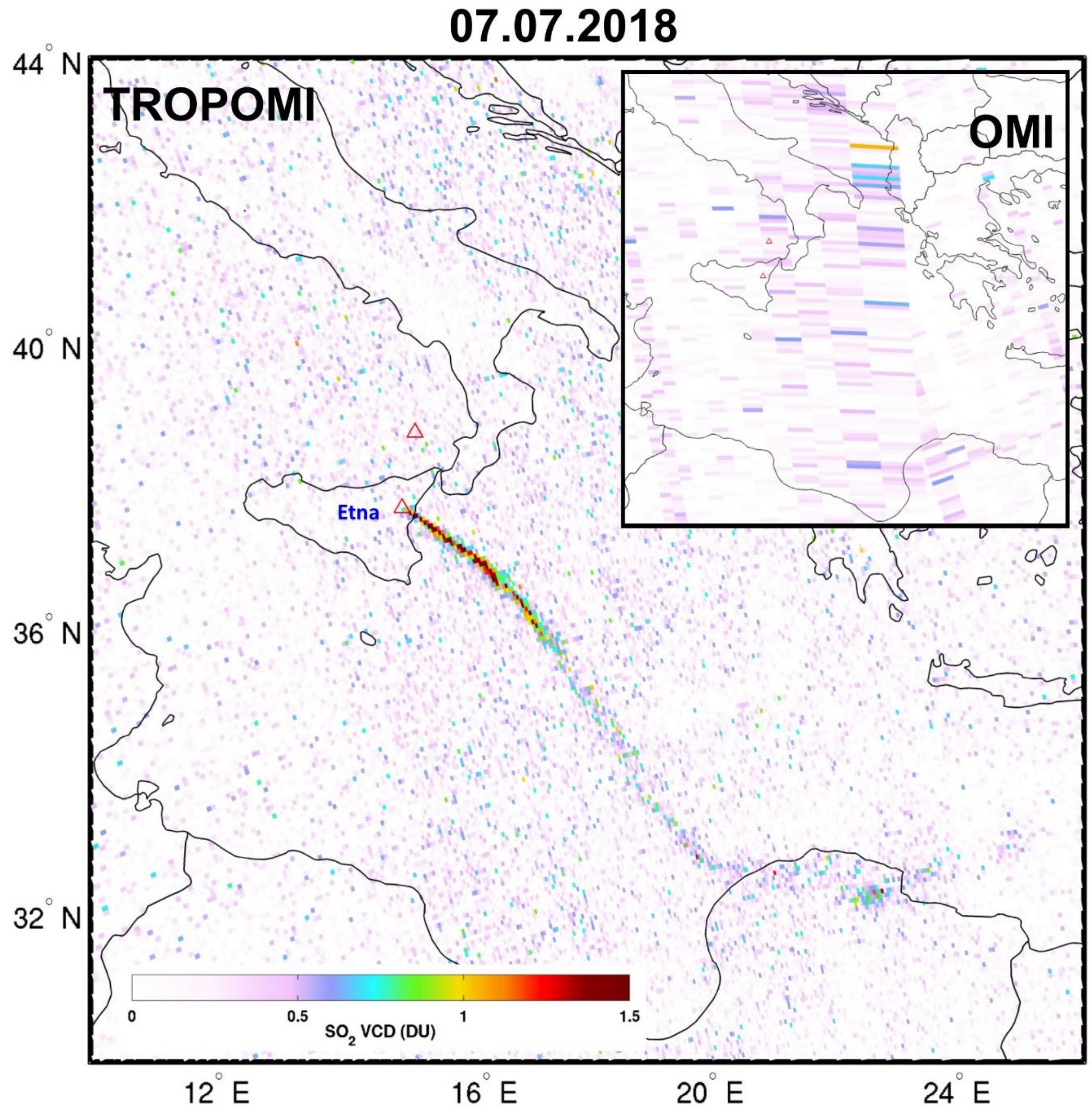

Figure S1. TROPOMI and OMI SO_2 vertical columns over the Mediterranean Sea on July 7, 2018, with SO_2 emission from Etna volcano.

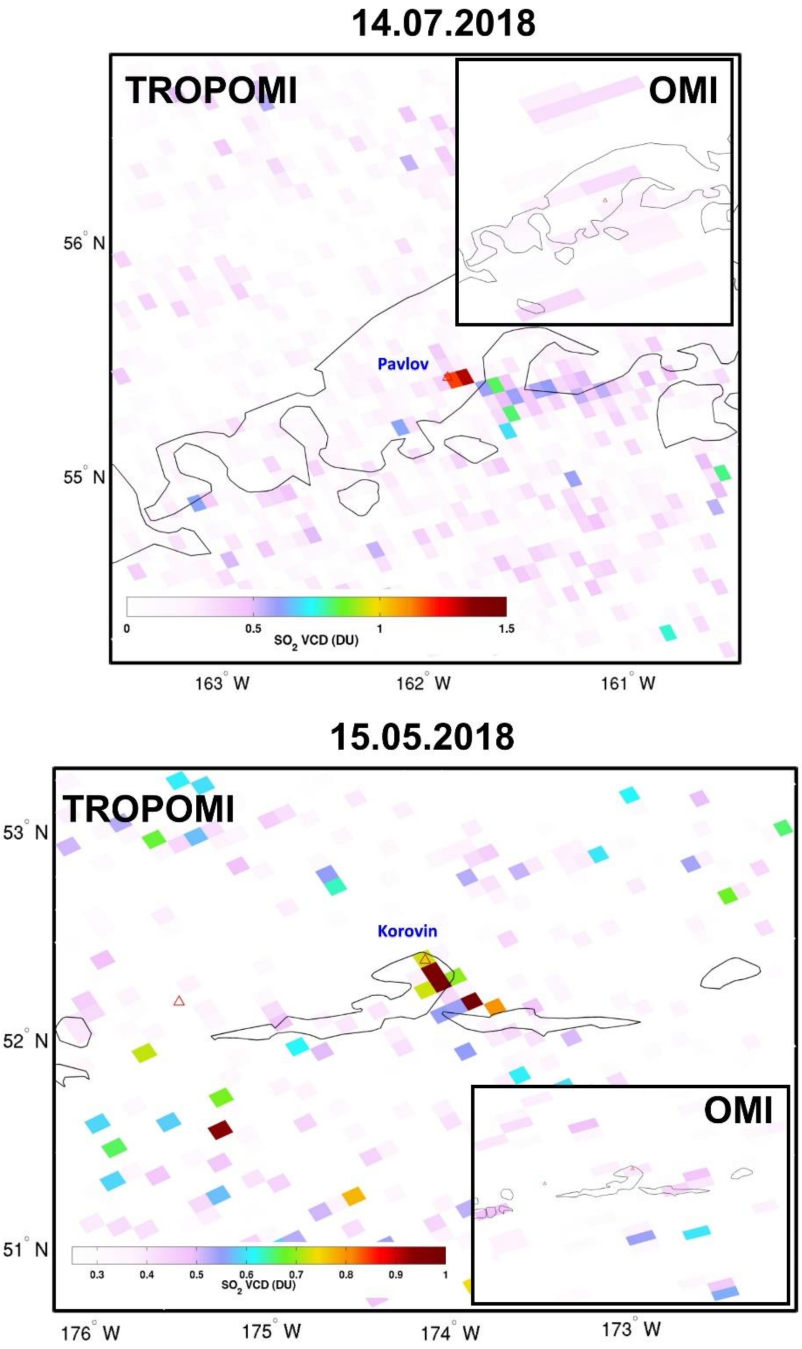

Figure S2. TROPOMI and OMI SO_2 vertical columns over the Aleutian Islands on July 14, 2018, with SO_2 emission from Pavlov volcano (top panel) and on May 15, 2018, with SO_2 emission from Korovin volcano (bottom panel).

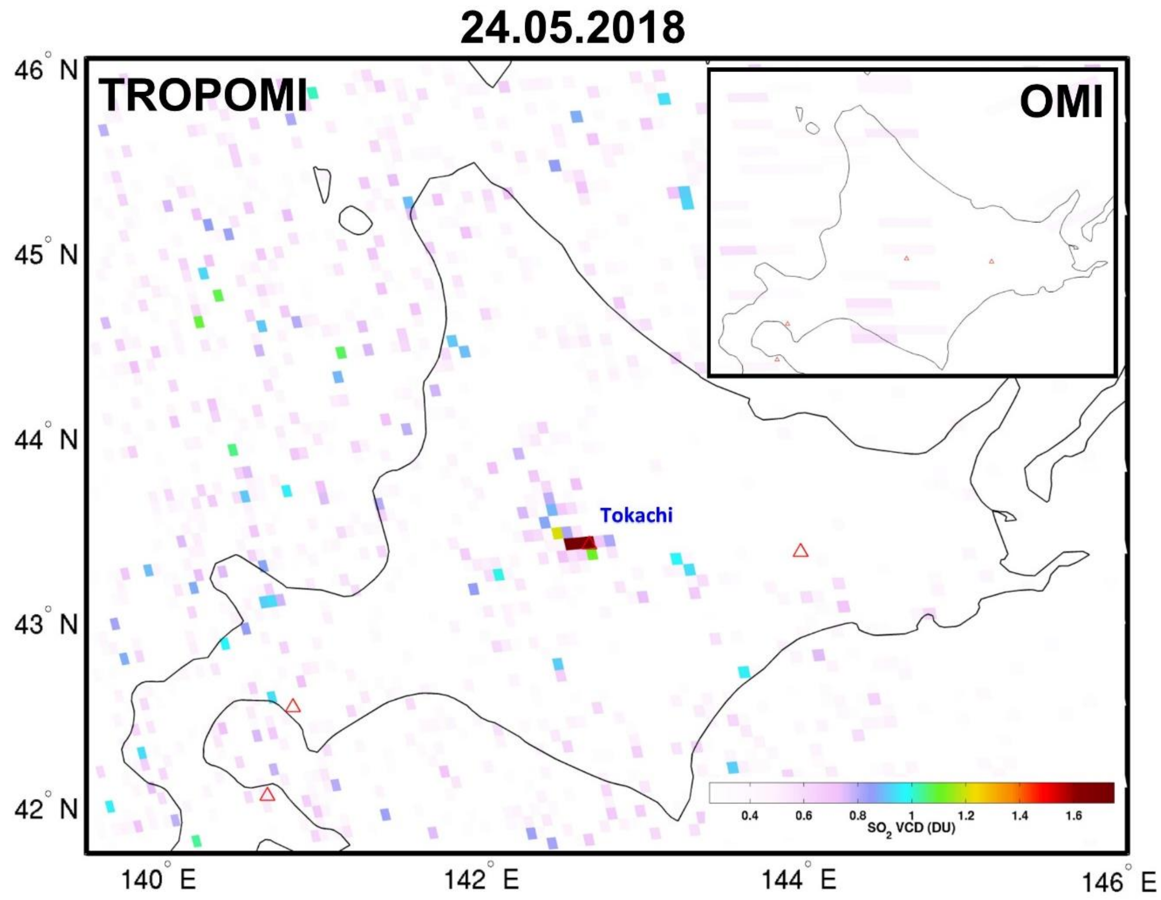

Figure S3. TROPOMI and OMI SO_2 vertical columns over the Hokkaido island of Japan on May 24, 2018, with SO_2 emission from Tokachi volcano.

References

1. Veefkind, J.P., Aben, I., McMullan, K., Förster, H., de Vries, J., Otter, G., Claas, J., Eskes, H.J., de Haan, J.F., Kleipool, Q., van Weele, M., Hasekamp, O., Hoogeven, R., Landgraf, J., Snel, R., Tol, P., Ingmann, P., Voors, R., Kruizinga, B., Vink, R., Visser, H., and Levelt, P.F. TROPOMI on the ESA Sentinel-5 Precursor: A GMES mission for global observations of the atmospheric composition for climate, air quality and ozone layer applications. *Remote Sensing of Environment*, doi:10.1016/j.rse.2011.09.027 (2012).
2. Levelt, P.F., van den Oord, G.H.J., Dobber, M.R., Mälkki, A., Visser, H., de Vries, J., Stammes, P., Lundell, J., and Saari, H. The Ozone Monitoring Instrument. *IEEE Trans. Geo. Rem. Sens.*, Vol. 44, No. 5, 1093-1101, doi:10.1109/TGRS.2006.872333 (2006).
3. Galle, B., Johansson, M., Rivera, C., Zhang, Y., Kihlman, M., Kern, C., Lehmann, T., Platt, U., Arellano, S., and Hidalgo, S. Network for Observation of Volcanic and Atmospheric Change (NOVAC)—A global network for volcanic gas monitoring: Network layout and instrument description. *J. Geophys. Res.*, 115, D05304, doi:10.1029/2009JD011823 (2010).
